# PPARγ Gene as a Possible Link between Acquired and Congenital Lipodystrophy and its Modulation by Dietary Fatty Acids

**DOI:** 10.3390/nu14224742

**Published:** 2022-11-10

**Authors:** Carmen Rodríguez-García, Cristina Sánchez-Quesada, María José Martínez-Ramírez, José J. Gaforio

**Affiliations:** 1 Department of Health Sciences, Faculty of Experimental Sciences, University of Jaén, 23071 Jaén, Spain; 2 University Institute of Research in Olive Groves and Olive Oils, University of Jaen, Campus las Lagunillas s/n, 23071 Jaén, Spain; 3 Agri-food Campus of International Excellence (ceiA3), 14071 Córdoba, Spain; 4 Endocrinology and Nutrition Clinical Management Unit, University Hospital of Jaén, 23007 Jaén, Spain; 5 CIBER Epidemiología y Salud Pública (CIBER-ESP), Instituto de Salud Carlos III, 28029 Madrid, Spain

**Keywords:** lipids, lipotoxicity, inflammation, metabolism, STRING

## Abstract

Lipodystrophy syndromes are rare diseases that could be of genetic or acquired origin. The main complication of lipodystrophy is the dysfunction of adipose tissue, which leads to an ectopic accumulation of triglycerides in tissues such as the liver, pancreas and skeletal muscle. This abnormal fat distribution is associated with hypertriglyceridemia, insulin resistance, liver steatosis, cardiomyopathies and chronic inflammation. Although the origin of acquired lipodystrophies remains unclear, patients show alterations in genes related to genetic lipodystrophy, suggesting that this disease could be improved or aggravated by orchestrating gene activity, for example by diet. Nowadays, the main reason for adipose tissue dysfunction is an imbalance in metabolism, caused in other pathologies associated with adipose tissue dysfunction by high-fat diets. However, not all dietary fats have the same health implications. Therefore, this article aims to summarize the main genes involved in the pathophysiology of lipodystrophy, identify connections between them and provide a systematic review of studies published between January 2017 and January 2022 of the dietary fats that can modulate the development of lipodystrophy through transcriptional regulation or the regulation of protein expression in adipocytes.

## 1. Introduction

Lipodystrophy syndromes are rare disorders characterized by adipose tissue (AT) dysfunction [1]. The dysfunction of adipocytes leads to immunometabolic complications such as chronic inflammation, ectopic fat accumulation, insulin resistance, hypoleptinemia, hypoadiponectinemia and hypertriglyceridemia [2]. These comorbidities should be managed to avoid the development of non-alcoholic fatty liver disease (NAFLD), diabetes mellitus type 2 (DMT2) and cardiomyopathies associated with lipodystrophy syndromes [3]. Lipodystrophy syndromes may be of congenital or acquired origin. Certain genes have been selected as the main contributors to different types of lipodystrophies [2]. However, in the case of acquired lipodystrophy, the origin remains unclear, despite the expression of specific genes, such as peroxisome proliferator-activated receptor gamma (PPARγ), being implicated in lipodystrophy associated with antiretroviral therapy (ART) [4].

Certain exogenous factors such as pharmacological or nutritional factors can modulate AT by orchestrating gene activity. Obesogenic diets, characterized by a high-fat composition, can downregulate certain gene expression in adipocytes that are related to adipogenesis and lipid metabolism alteration [5]. However, dietary fats have different metabolic targets based on their composition in terms of fatty acids and not all have the same effects on health [6]. Therefore, the aim of this article was to summarize the main genes involved in the pathophysiology of lipodystrophy, to identify the connection between them and to analyze in a systematic review the dietary fats that can modulate the development of lipodystrophy through transcriptional regulation or the regulation of protein expression in adipocytes.

## 2. Materials and Methods

The methodology has been divided into:

1. Initial research on the most common types of lipodystrophies and the main genes involved in the development of congenital lipodystrophy as well as the genes implicated in acquired lipodystrophy.
2. Analysis of the proteins involved in lipodystrophy via the Search Tool for the Retrieval of Interacting Proteins 11 (STRING 11) (a database under a Creative Commons by 4.0’ license) with a minimum required interaction score (high confidence (0.700)) [7]. This analysis was performed to determine the interaction between the different proteins implicated in lipodystrophy and to identify which ones played a more relevant role and the main biological processes in which they were involved. In addition, it allowed us to determine the relationship between the implicated proteins in congenital and acquired lipodystrophy.
3. A systematic review of the scientific evidence on the modulation of the expression and activity of the selected genes (PPARγ and Perilipin 1 (PLIN1)) by dietary lipids. The PubMed database was searched from January 2017 to January 2022. Due to the lack of studies analyzing the impact of diet on lipodystrophy, the following search strategy was used: ((PPARG) OR (peroxisome proliferator-activated receptor-gamma) OR (PLIN1) OR (PERILIPIN1)) AND ((oil) OR (fatty acid) OR (high-fat diet) OR (dietary lipid) OR (capric acid) OR (lauric acid) OR (myristic acid) OR (palmitic acid) OR (stearic acid) OR (arachidic acid) OR (behenic acid) OR (caprylic acid) OR (oleic acid) OR (linoleic acid) OR (eicosapentaenoic acid) OR (linolenic acid) OR (arachidonic acid) OR (docosatetraenoic acid) OR (palmitoleic acid)) NOT (review [Publication Type]). The inclusion criteria were as follows: (1) experimental model: cells, mice, rats and clinical trials; (2) intervention with dietary oils, fats or isolated fatty acids (i.e., conjugated linoleic, palm oil or omega-3 fatty acids); (3) analysis of PPARγ or PLIN1 gene/protein expression; (4) original papers (not reviews); (5) articles written in the English language. The eligibility for inclusion and exclusion criteria were evaluated by reading both (1) the title and abstract and (2) the full text (Supplementary Figure S1).

## 3. Results and Discussion

### 3.1. Lipodystrophy Syndromes Classification

Lipodystrophy syndromes are disorders characterized by a redistribution of AT. They affect either localized areas (partial) or the whole body (generalized) [8]. Lipodystrophy may appear as an undesirable effect of certain drugs (i.e., insulin, antiretroviral therapies, etc.), due to autoimmune mechanisms, or has a genetic origin (autosomal dominant or recessive subtypes) [1]. Acquired lipodystrophy occurs with metabolic syndromes, human immunodeficiency virus (HIV), connective tissue disorders and some inflammatory conditions.

**Figure 1:**
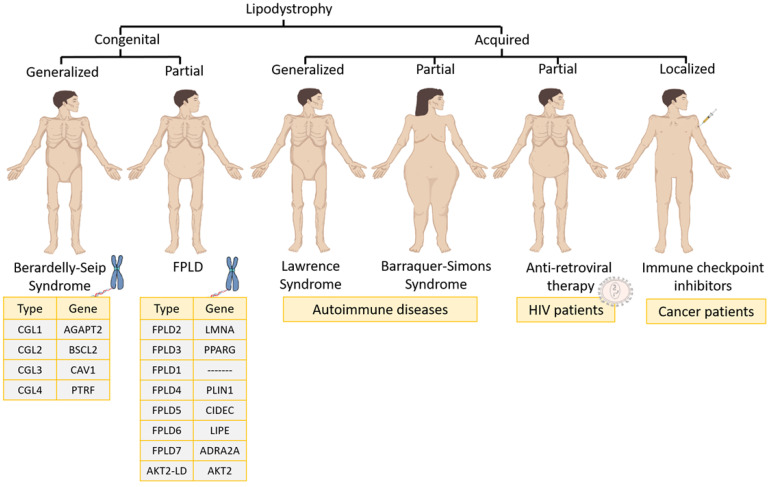
Lipodystrophy syndromes classification. FPLD, familial partial lipodystrophy.

The different types of lipodystrophy syndromes can be classified as follows (Figure 1):

#### 3.1.1. Congenital Lipodystrophies

- Familial partial lipodystrophy (FPLD) is usually an autosomal dominant syndrome characterized by a selective loss of fat from the lower and upper extremities as well as the trunk [1]. During childhood, patients have a normal fat distribution but at puberty start to lose fat from the chest, anterior abdomen and extremities. There are eight varieties of FPLD: (1) FLPD2, the most common subtype (also called the Dunnigan type), which is characterized by mutations in the lamin A/C gene (LMNA); (2) FPLD3, the second most common subtype, which is based on mutations in the PPARγ gene; (3) FPLD1 (or the Kobberling type), whose genetic mutation is unknown; (4) FPDL4, which is characterized by heterozygous mutations in the PLIN1 gene; (5) FPDL5 and (6) FPDL6, both of which are autosomal recessive disorders in the cell death-inducing DFFA-like effector C (CIDEC) and lipase E (LIPE) genes, respectively; (7) FPDL7, which features a genetic mutation in the adrenoceptor alpha 2A (ADRA2A) gene; and (8) AKT2-linked lipodystrophy (AKT2-LD), which is based on a mutation in the (AKT Serine/Threonine Kinase 2) AKT2 gene [1].
- Congenital generalized lipodystrophy (CGL), or Berardinelli–Seip syndrome, is an autosomal recessive disease that is distinguished by the absence of AT both at birth and in early childhood [1]. Four distinct subtypes exist depending on the gene that is altered: (1) CGL1 is the most common subtype, and the associated altered gene is 1-Acylglycerol-3-Phosphate O-Acyltransferase 2 (AGPAT2), which is involved in triglyceride biosynthesis; (2) CGL2 is the second most common subtype and the altered gene in that case is lipid droplet biogenesis associated (BSCL2), which plays a relevant role in adipocyte differentiation and small lipid droplet fusion in adipocytes; (3) CGL3 has only been reported in one patient and the altered gene was caveolin 1 (CAV1), which translocates fatty acids to lipid droplets; (4) CGL4 has been reported in 20 patients and is very close to CGL3 because its gene (caveolae-associated protein 1) is regulated by CAV1 expression [1].

#### 3.1.2. Acquired Lipodystrophies

- Acquired generalized lipodystrophy or Lawrence syndrome is characterized by the generalized loss of subcutaneous fat. The loss of fat usually begins in childhood or adolescence [1]. Most patients have related autoimmune diseases [8,9,10,11,12,13]. In certain autoimmune diseases, the role of the PPARγ gene is essential to modulate inflammation. In fact, therapy is based on agonists of the PPARγ gene [14].
- Acquired partial lipodystrophy, or Barraquer–Simons syndrome, is characterized by a gradual loss of subcutaneous fat from the upper trunk, upper extremities, neck and face. In the case of females, after puberty, excess fat can accumulate in the lower extremities, hips and lower abdomen. It is often related to autoimmune diseases and this syndrome affects mostly women [1,15,16,17].
- Antiretroviral therapy-induced lipodystrophy occurs in patients infected with human immunodeficiency virus who, after 2–4 years of treatment with ART, start to have an increased accumulation of both intra-abdominal and upper trunk fat, while they lose subcutaneous fat in the lower and upper extremities [1,18]. This is related to PPARγ protein downregulation. However, by stopping ART, PPARγ protein expression is restored in macrophages and adipocytes [4].
- Recent cases of acquired lipodystrophy have been associated with the use of immune checkpoint inhibitors to treat cancer [19,20,21]. Among them, childhood cancer survivors transplanted with hematopoietic stem cells and treated with chemotherapy developed acquired lipodystrophy over time [22]. Furthermore, other types of cancer, such as craniopharyngioma, may lead to chronic inflammatory demyelinating polyneuropathy together with acquired lipodystrophy [23]. This pathology is associated with certain cancer therapies against programmed cell death protein 1 (PD-1), which promotes apoptosis in antigen-specific T cells in lymph nodes and leads to adverse events of an immune nature [24].

### 3.2. Relationship of Genes Involved in the Development of Lipodystrophy

The main genes involved in the development of lipodystrophy of congenital origin and those implicated in specific cases of acquired lipodystrophy are summarized below:

- BSCL2: a protein expressed mainly in AT, which is involved in lipid droplet biogenesis, in the regulation of energy homeostasis and adipocyte differentiation [25].
- LIPE or hormone-sensitive lipase, which promotes the hydrolysis of triglycerides stored in lipid droplets during adipocyte differentiation [26].
- CAV1: a protein located in lipid droplets of adipocytes which has a key role in cholesterol homeostasis, endothelial transcytosis and cellular metabolism [27].
- LMNA: a protein involved in telomere dynamics, the nuclear membrane, chromatin organization and nuclear assembly [28].
- AKT2: a kinase involved in processes such as angiogenesis, cell growth, proliferation and metabolism [29].
- ADRA2A: a receptor involved in the inhibition of adenylate cyclase induced by catecholamine [30].
- PPARγ: a nuclear receptor that controls insulin sensitivity, glucose metabolism and adipocyte differentiation. PPARγ protein is a major adipogenic factor [31].
- AGPAT2: an acyltransferase involved in the transformation of lysophosphatidic acid into phosphatidic acid, which belongs to the triglyceride biosynthetic pathway [32].
- CIDEC: a protein that modulates triglyceride storage by restricting lipolysis and is involved in the enlargement of lipid droplets [31].
- PLIN1: a modulator of the lipid metabolism in adipocytes which protects lipid droplets from breakdown by HSL, and its interaction with CIDEC promotes the enlargement of lipid droplets [33].

**Figure 2:**
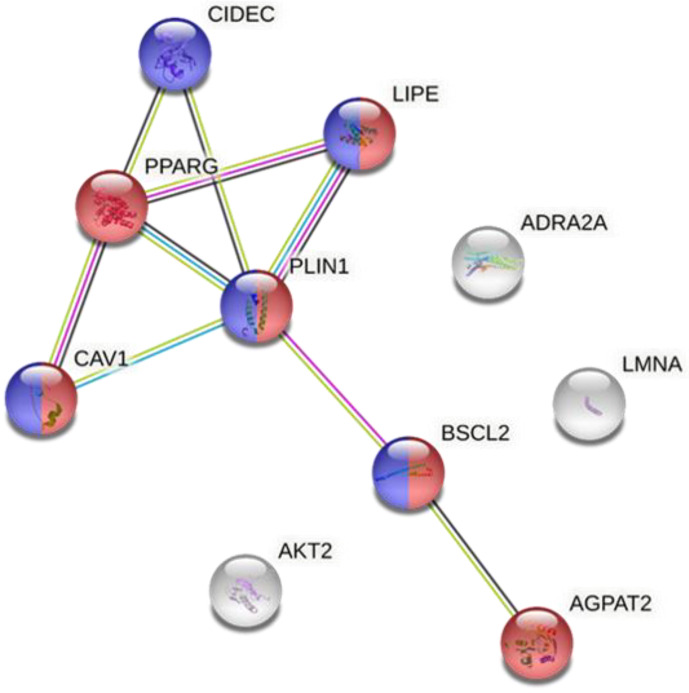
Protein–protein interaction network. Colored nodes in red: proteins involved in lipid metabolism. Colored nodes in blue: proteins involved in lipid droplet formation. Colored nodes in white: the second shell of interactions. Edges represent protein–protein associations. Associations are meant to be specific and meaningful, i.e., proteins jointly contribute to a shared function; this does not necessarily mean that they are physically binding to each other. Depending on the color of the line, the association has been determined from curated databases (blue line) or experimentally (pink line). If the genes are neighbors, the interaction line is green and if the genes are co-expressed, the lines are black. The more interaction lines between two genes, the stronger the evidence of their interaction.

STRING11 software was used to analyze if there was any relationships between the main proteins implicated in the different types of lipodystrophies. The results of the analysis showed that there was an interaction between certain proteins: PPARγ, CIDEC, CAV1, LIPE, PLIN1, BSCL2 and AGPAT2 (Figure 2). We observed that there were strong interactions between PPARγ and PLIN1 and most proteins. In fact, the network had significantly more interactions than expected, which means that proteins have more interactions among themselves than what would be expected from a random set of proteins, demonstrating that the proteins are partially biologically connected as a group. This group is mainly involved in biological processes that are closely related to lipodystrophy such as the lipid metabolism and lipid droplet formation. Thus, the data suggested that PPARγ and PLIN1 proteins can play an important role in the dysfunction of adipose tissue by modulating the activity of other proteins. Surprisingly, PPARγ protein expression is also altered in specific types of acquired lipodystrophy, which could be the link between congenital and acquired lipodystrophy and a key target for the management of the disease. Therefore, the next step was to identify how the expression of both proteins can be modulated by nutritional factors such as dietary fatty acids.

### 3.3. Nutrigenomic Effects of Dietary Lipids on PPARγ and PLIN1

Gene regulation in AT can be produced by diet. Dietary fat composition can affect ectopic lipid accumulation and dietary fatty acids are involved in the composition of cellular membranes, organelles membranes and can regulate toll-like receptor (TLR) activity, contributing to the inflammatory response [34,35]. However, studies that have analyzed the effect of dietary lipids on lipodystrophy are limited. After a review of the scientific literature, 37 articles were selected for discussion in this section (Supplementary Figure S1).

Interventions with oils have been developed primarily in murine models, although there are clinical trials on diabetic and healthy adults (Table 1). Patients with metabolic dysregulation such as DMT2 have common complications such as cardiomyopathy, a typical outcome in certain lipodystrophic patients [36]. A study analyzed the effect of Krill oil (an oil rich in n-3 polyunsaturated acid (PUFA) of marine origin) in the prevention of cardiomyopathy in diabetic mice [37]. This oil was able to upregulate the peroxisome proliferator-activated receptor-γ coactivator 1α (PGC-1α), which is involved in the inhibition of the inflammasome (NLR family pyrin domain containing 3 (NLRP3)). PGC-1α is a transcriptional coactivator that interacts with PPARγ and primarily regulates genes involved in energy metabolism (e.g., mitochondrial biogenesis). However, this transcriptional network mainly modulates the key signaling pathways of the production and differentiation of white and brown adipocytes [38]. In the same way, supplementation with flaxseed oil (rich in n-3 PUFAs) in diabetic patients with coronary heart disease significantly downregulated the expression of tumor necrosis factor alpha (TNF-α) and upregulated PPARγ protein in the peripheral blood mononuclear cells (PBMCs) of patients [39]. Supplementation with oil rich in n-3 PUFA was closely related to the fatty acid composition of different tissues and positively regulated the expression of PPARγ protein in the AT of turkeys, regulating fat metabolism [40]. An oils’ composition is directly associated with its impact on health. In the case of palm oil or butter, which is composed mainly of saturated fatty acids, these are related to the development of NAFLD, while the substitution of these oils with others rich in unsaturated fatty acids such as rapeseed oil can attenuate the progression of NAFLD by reducing the levels of lipopolysaccharide, downregulating the activation of TLR4 and increasing PPARγ activity in the small intestine [41]. While peanut oil and lard induced inflammation, hepatic steatosis and high blood pressure, a blended oil rich in oleic acid and ALA was able to reduce low-density lipoprotein cholesterol (LDL), serum triglycerides, TLR4 expression, TNF-α and C-reactive protein but increased PPARγ protein expression. These results demonstrate that an appropriate ratio of monounsaturated and n-6/n-3 PUFAs could prevent immunometabolic disturbances [42].

Virgin olive oil and fish oil have been demonstrated to be one of the healthiest fat sources in terms of maintaining body weight, regulating metabolism and promoting an anti-inflammatory status. However, there is no evidence of PPARγ activity modulation by virgin olive oil using the above search strategy. Regarding fish oil, its supplementation can significantly enhance lipoprotein lipase, PPARγ and PGC-1α protein expression, even during a high-fat diet intervention in rats [43]. Supplementation with fish oil enriched in docosahexaenoic acid (DHA) in diabetic patients increased PPARγ activity significantly in PBMCs, improving patient metabolism [44].

On the other hand, palm oil and palmitic acid-induced lipotoxicity in the liver enhances hepatic fatty acid and triglyceride uptake by the upregulation of CD36, a very-low-density lipoprotein receptor (VLDLR), and PPARγ protein [45]. Despite their different effects on health, overfeeding promotes the methylation of PGC-1α and TNF-α in AT [46].

Regarding studies that analyze the effect of n-3 PUFAs in different research models (Table 2), all conclude that n-3 PUFAS can suppress metabolic disturbances associated with insulin resistance and the development of liver fibrosis. In the white adipose tissue of rats, these fatty acids induce browning by enhancing the activity and expression of PPARγ protein and enhancing the expression of neuregulin 4 (Nrg4), which is involved in the prevention of lipid accumulation in hepatic cells [47]. Furthermore, in murine models of metabolic syndrome, n-3 PUFAs supplementation increased the expression of PPARγ and glucose transporter type 4 (GLUT4) proteins, improving insulin sensitivity and the lipid profile [48]. Similarly, supplementation with n-3 PUFAs in athletes produced an upregulation of the protein levels of uncoupling protein 2 (UCP2) and PPARγ proteins in PBMCs, improving energy expenditure and controlling body weight [49].

Based on the protective effects of n-3 PUFAs supplementation, several studies have individually analyzed the effects of EPA and DHA to elucidate their mechanism of action (Table 3). Both EPA and DHA fatty acids have shown an anti-lipotoxic effect in different types of cells. In a high-fat diet or during the induction of lipotoxicity with palmitic acid in vitro, supplementation with EPA was able to promote fatty acid oxidation and lipid droplet formation [54]. Similarly, supplementation in obese mice attenuated the dysfunction of AT by enhancing the expression of PPAR-γ protein and reducing the inflammation associated with high levels of interleukin-6 (IL-6) and TNFα, attenuating the inflammatory-metabolic state [55]. Furthermore, in UCP1 knockout mice fed a high-fat diet, who are unable to regulate diet-induced thermogenesis and are at increased risk for obesity, EPA supplementation exerted protective effects, increasing PGC1α expression in brown adipose tissue and improving glucose tolerance [56]. Treatment with EPA and DHA, alone or combined, upregulated different genes involved in the mitochondrial function. In skeletal muscle cells, EPA improved the response to insulin via PGC1-α and countered the inflammation induced by palmitic acid, inhibiting the nuclear factor kappa-light-chain-enhancer of activated B cell (NFκB) signaling [57]. In the same way, EPA promotes adipogenesis in mesenchymal stem cells through the activation of PPARγ, while treatment with EPA, DHA or furan fatty acid 9-(3-methyl-5-pentylfuran-2-yl)-nonanoic acid (9M5) induced adipogenesis in preadipocytes [58,59,60]. Comparing the effects of EPA and DHA, the latter has been demonstrated to be more effective in immunometabolic regulation, enhancing fat oxidation in muscle cells (via PGC1-α), reducing the expression of TNFαR, improving adipocyte functionality through PPARγ activity and increasing adiponectin secretion [61,62]. In combination with arachidonic acid (ARA), DHA attenuated the AT dysfunction induced by an obesogenic diet and can reduce inflammatory cytokines such as TNF-α IL-6 in AT [63].

The effects of conjugated linoleic acid (CLA) on the metabolism remain controversial (Table 4). Its supplementation has been shown to decrease the abundance of genes related to fatty acid oxidation and lipolysis and to increase genes involved in lipogenesis, such as the PPARγ gene [64]. In murine models, high doses of CLA are associated with lipid accumulation in the liver by negatively regulating PCG-1α, leading to steatogenic effects as well as a reduction in body fat through the modulation of PPARγ protein [65,66]. Contrary to the effects of n-3 PUFAS, CLA does not stimulate mitochondrial biogenesis or PCG-1α expression in murine models [67]. On the other hand, the in vitro effects of CLA in combination with alpha-lipoic acid exerted anti-inflammatory activity in murine macrophages through the modulation of the ERK1/PPARγ pathway [68].

**Figure 3:**
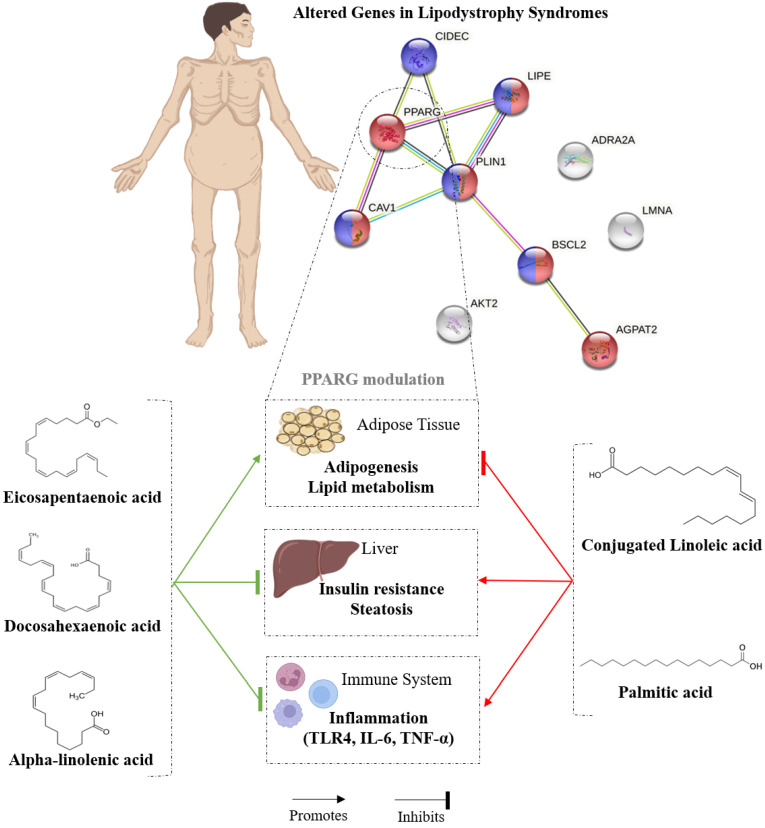
PPARγ gene modulation by dietary fatty acids.

Overall, these results suggest that while SFA and CLA induce dysfunction in AT and negatively regulate adipogenesis and lipid metabolism, n-3 PUFAs and certain monounsaturated fatty acids can reverse the effects caused by saturated fatty acids and a high-fat diet (Figure 3).

However, there are important aspects to highlight for future analysis in both the diagnosis of congenital lipodystrophy and in the approach to genetic alterations through diet. On the one hand, the alterations in PPARs expression in patients only reflect the alteration in the PPARγ isoform, and there are no data on the PPARα and PPARβ/δ isoforms that are also involved in adipocyte metabolism. On the other hand, it would be interesting to analyze if alterations in PPARγ are a consequence and not a cause of adipose tissue atrophy. Lipodystrophic adipose tissue is characterized by both a high infiltration of M1 (proinflammatory phenotype) and a reduction in resident M2 macrophages. It has been observed that PPARγ is also involved in the polarization toward M2 phenotype macrophages, so their downregulation in adipose tissue could be the cause of the decrease in PPARγ expression. However, further studies are needed to elucidate the mechanism of lipodystrophy development. Furthermore, in order to obtain results that can be extrapolated to clinical practice, the effect of dietary fats and oils on the modulation of adipose tissue function in patients with lipodystrophy needs to be analyzed in clinical trials.

## 4. Conclusions

Congenital and acquired lipodystrophy share a common pathophysiology: adipose tissue dysfunction, an alteration that results in most cases in hypertriglyceridemia, ectopic fat accumulation, insulin resistance, chronic inflammation and low levels of leptin and adiponectin. Consequently, a low-fat diet is usually recommended to lipodystrophy patients, but not all fats have harmful effects on the health of these patients. In this article, we determined a common link through genetic alteration in specific congenital and acquired lipodystrophies. We observed that PPARγ is a gene involved in lipodystrophy development and is closely associated with the regulation of the other genes altered in this pathology. The PPARγ gene may be up-regulated in adipose tissue by omega-3 polyunsaturated fatty acids such as EPA, DHA and ALA, while saturated fatty acids such as palmitic acid lead to PPARγ up-regulation in non-adipose tissues such as the liver, favouring ectopic fat accumulation. Omega-3 may prevent or reduce the typical comorbidities of lipodystrophy such as type 2 diabetes mellitus, NAFLD and cardiomyopathies, improving the symptomatology of lipodystrophy, whereas saturated fatty acids may worsen these comorbidities. However, more clinical studies are required to determine the role of dietary fat in PPAR-γ gene modulation to control adipose tissue dysfunction in lipodystrophic patients.

## Figures and Tables

**Table 1:** Effect of oils on PPARγ modulation.

	Intervention	Duration	Study Design	Outcome	References
Krill oil (KO)	AING-93 G diet for control and 1.5% KO-containing high-fat diet	24 weeks	In vivo Diabetic C57BL/6 mice	KO increases cardiac PGC1-α protein expression in cardiac tissue, reducing apoptosis of cardiomyocytes and negatively regulating the NRLP3 inflammasome	[37]
Flaxseed Oil	Placebo or 1000 mg flaxseed oil supplements (400 mg α-linolenic acid) (twice a day)	12 weeks	Randomized double-blind placebo-controlled trial Diabetic patients with coronary heart disease (40–85 years) *n* = 60	Flaxseed oil supplementation up-regulates PPARγ gene expression and reduces TNF-α levels	[39]
Linseed oil (LO)	A basic diet or a basic diet supplemented with 0.5% or 1% of LO	22 weeks	In vivo *Meleagris gallopavo* *n* = 140	LO supplementation leads to higher levels of PPARγ in adipose tissue	[40]
Rapeseed oil	Control diets with 12% of energy from soybean oil or 25% of energy from butterfat. Diet with 12% of energy from rapeseed oil or a diet with rapeseed oil, fructose and cholesterol	13 weeks	In vivo C57Bl/6J mice with NAFLD *n* = 28	Rapeseed oil exerts protective effects against NAFLD by increasing PPARγ activity, lowering portal endotoxin levels and attenuating TLR4 signal	[41]
Blended oil	Low-fat, middle. A fat and high-fat diet based on peanut oil, lard oil diet or blended oil (canola oil, corn oil, olive oil, peanut oil, sunflower oil)	12 weeks	In vivo Wistar rats *n* = 90	A blended oil diet enhances genes related to lipid catabolism such as PPARγ in the liver, due mainly to their n-6/n-3 PUFAs ratio. Blended oil reduces TNF-α and C-reactive protein in serum	[42]
Fish oil	High-fat diet or a high-fat diet with 5% of fish oil (30.6% of EPA and 19.2% of DHA)	8 weeks	In vivo Sprague-Dawley rats *n* = 18	High-fat diet in fish oil may reverse the protein expression in soleus muscle of PPARγ and PGC-1α protein, improving lipid metabolism	[43]
DHA-enriched fish oil	Soft gels of 2400 mg of fish oil with 600 mg of n-3 PUFAs or paraffin oil. Four gels per day	8 weeks	A double-blind randomized controlled trial Patients with DMT2 *n* = 50	DHA-rich fish oil increases PPAR-γ activity in PBMCs	[44]
Palm oil	Low-fat diet or a high-fat diet based on palm oil (60%)	12 weeks	In vivo C57BL6 mice *n* = 32	A high-fat diet based on palm oil increases the hepatic expression of VLDLR, CD36 and PPARγ, leading to excess hepatic lipid accumulation	[45]
Palm oil and sunflower oil	High-calorie muffins that contain refined sunflower oil or refined palm oil.	7 weeks	A double-blind randomized controlled trial Healthy adults (20–38 years) *n* = 31	After overfeeding, the methylation of PGC-1α and TNF-α in adipose tissue was increased	[46]

DHA: docosahexaenoic acid; EPA: eicosapentaenoic acid; KO: krill oil; LO: linseed oil; NAFLD: non-alcoholic fatty liver disease; PUFA: polyunsaturated acid.

**Table 2:** Effect of n-3 PUFAS on PPARγ modulation.

	Intervention	Duration	Study Design	Outcome	References
n-3 PUFAs	A high-fat diet rich in saturated fats or n-3 PUFAs or a normal diet.	13 weeks	In vivo male Sprague-Dawley *n* = 30	n-3 PUFAs induce browning in white adipose tissue by the PPARγ pathway, and EPA increases PPARγ in HPAs.	[47]
	0, 5, 10 and 20 μmol/L of EPA	Data not shown	In vitro Human preadipocytes		
n-3 PUFAs	Chow diet, high-fat diet + 260 mg/kg n-3 PUFA, high-fat diet + 100 mg/kg metformin or high-fat diet + 200 mg/kg L-carnitine	12 weeks	In vivo Sprague Dawley rats *n* = 30	n-3 PUFAs decrease body weight, glucose and insulin and increase adiponectin by GLUT4 and PPARγ regulation	[48]
n-3 PUFA	Supplement of two Omega 3 gel capsules per day (240 mg of DHA and 360 mg EPA) or placebo (paraffin oil capsules)	3 weeks	A double-blind randomized controlled trial Peripheral blood mononuclear cells of athletes (PBMCs) *n* = 36	Omega 3 supplementation leads to the up-regulation of PPARγ protein levels in the blood	[49]
n-3 PUFAs	Normocaloric diet or a high-fat diet supplemented with metformin, 300 mg/kg/d of n-3 PUFAs or a combination of both.	8 weeks	In vivo diabetic Sprague-Dawley rats *n* = 38	n-3 PUFAs alone or in combination with metformin can suppress metabolic changes related to diabetes by increasing the gene expression of PGC-1α	[50]
n-3 PUFAS	Four capsules/day of 0.5 g of n-3 PUFAs from fish oil, 0.62 g of α-linolenic acid from flaxseed and corn oil as a control	180 days	A double-blind randomized controlled trial Patients with DMT2 (35–80 years) *n* = 185	T2D patients with different genotypes at PPARγ, NOS3 and CD36 respond differentially to the intervention of omega-3 supplements in blood lipid profiles	[51]
n-3 PUFAs	Treatment with palmitic acid, oleic acid, linoleic acid, ALA, EPA and DHA at 50 μM	24 h	In vitro LX-2 cells Primary human and mouse hepatic stellate cells	α-linolenic acid, EPA and DHA prevent liver fibrosis development by PPARγ regulation	[52]
n-6:n-3 PUFAs	0.1, 0.2 and 0.4 μg/mL of chia seed extract fatty acid with a ratio of 3:1 of omega 3 and omega 6	14 days	In vitro Human bone marrow-derived mesenchymal stem cells PBMCs from healthy volunteer	Chia seed extracts fatty acid, decreases lipid accumulation and enhances mitochondrial fatty acid oxidation in mature adipocytes by increasing the expression of PGC-1α. Furthermore, this treatment suppresses macrophage recruitment in adipocytes and reduces protein levels of TNF-α	[53]

ALA: α- linolenic acid; DHA: docosahexaenoic acid; EPA: eicosapentaenoic acid; PBMCs: peripheral blood mononuclear cells; PUFA: polyunsaturated fatty acids. Drug therapy for patients with DMT2 includes the use of drugs such as metformin, which improves insulin sensitivity in tissues such as the liver and skeletal muscle. N-3 PUFAs supplementation alone or in combination with metformin restores metabolism by increasing the PGC-1α expression, preventing mitochondrial degeneration, having a hypoglycemic effect, and improving the myocardial structure to prevent cardiomyopathies in rats [50]. Depending on the genes altered in DMT2, the response to n-3 PUFA supplementation may vary. However, n-3 PUFAs can modulate the expression of genes involved in metabolism maintenance such as the PPARγ gene, nitric oxide synthase 3 (NOS3) and CD36, improving the lipid profile of patients [51]. During insulin resistance, the liver is one of the most damaged organs due to the excessive accumulation of triglycerides. N-3 PUFAs can prevent liver fibrosis in hepatic cells, due mainly to their agonist effect on PPARγ protein, being more potent in the treatment with DHA and EPA than ALA [52]. Despite the health benefits of other PUFAs such as n-6, the amount of n-3 to n-6 in the diet should be a 3:1 ratio to control the macrophage recruitment to adipocytes during AT dysfunction, to improve fatty acid oxidation and to reduce inflammation via TLR4 and TNF-α [53].

**Table 3:** Effect of eicosapentaenoic acid, docosahexaenoic acid and arachidonic acid on PPARγ and PLIN1 modulation.

	Intervention	Duration	Study Design	Outcome	References
EPA	Normocaloric diet, high-fat diet or high-fat diet mixed with 3.6% of EPA (weight/weight)	8 weeks	In vivo GFP-MAP1LC3 transgenic, atg5-cKO (Atg5^F/F^; Kap-Cre) and atg5-iKO (Atg5^F/F^; Ndrg1-Cre) mice *n* = 21	EPA promotes lipid droplet formation and the transfer of fatty acid from them to the mitochondria for beta-oxidation, attenuating the lipotoxicity induced by a high-fat diet	[54]
EPA	Control diet or a high-fat diet supplemented with saline, EPA (50 mg/kg) or hidroxytyrosol (5 mg/kg) or a combination of both.	12 weeks	In vivo C57BL/6J mice *n* = 80	Supplementation with EPA improved the activity and mRNA levels of PPAR-γ and reduced the levels of TNFα and IL-6 in white adipose tissue	[55]
EPA	High-fat diet or a high-fat diet supplemented with 36 g/kg of EPA-enriched fish oil	14 weeks	In vivo Wild type and UCP1 knockout C57BL/6J mice *n* = 40	EPA can rescue glucose tolerance in UCP1 knockout mice via PGC1-α	[56]
EPA	500 μM of palmitic acid, 500 μM of EPA or 500 μM of palmitic acid combined with 100 μM of EPA	24 h	In vitro Human primary myotubes Human primary myoblasts derived from the abdominal rectus muscles of male individuals 31.00 ± 5.67 years	EPA increases the expression of PGC1-α and is coupled with the inhibition of the inflammatory response induced by palmitic acid.	[57]
EPA	EPA, DHA, linoleic acid or α-linolenic acid at 1, 10 or 100 μM	6, 24, 48 and 72 h	In vitro Primary cultures of gilthead sea bream bone-derived MSCs	EPA treatment up-regulates the genes involved in adipogenesis such as PPAR-γ	[58]
EPA/DHA	10 μM of EPA, 50 μM of DHA or 100 μM of EPA + 50 μM of DHA	24 h	In vitro Murine 3T3-L1 cell line	EPA and DHA, alone or combined, modulate the adipogenesis of adipocytes via PPARγ-CIDEC suppression	[59]
EPA/DHA	10 mM of DHA or 10 mM of EPA	24, 72 or 120 h	In vitro Rat L6 skeletal muscle cells	DHA increases the expression of PGC1-α, regulating metabolism and fat oxidation more effectively than EPA, and reduces the expression of TNFαR	[61]
EPA/DHA	EPA and DHA at 25, 50, 100 or 200 μmol/L	24 h	In vitro 3T3-L1 mouse preadipocytes	DHA led to an increase in PPARγ expression and secretes adiponectin at relatively low concentrations	[62]
DHA + ARA	A low-fat diet, high-fat diet, high-fat diet supplemented with ARA + DHA, high-fat diet supplemented with eHC or high-fat diet supplemented with ARA + DHA + eHC	12 weeks	In vivo Ucp1-2A-luciferase knock-in C57BL/6J *n* = 60	ARA + DHA supplementation improves metabolic flexibility and attenuates adipose tissue dysfunction during a high-fat diet as well as systemic inflammation-reducing IL-6 and TNF-α in adipose tissue	[63]
EPA/DHA/9M5	Treatment with 10 μM of 9M5, 50 μM of EPA or 50 μM of DHA alone or in different combinations.	48 h	In vitro 3T3-L1 preadipocytes	9M5 increases the protein expression of PPARγ and lipid accumulation during the differentiation process of 3T3-L1 preadipocytes into adipocytes	[60]

ARA: arachidonic acid; DHA: docosahexaenoic acid; eHC: extensively hydrolyzed casein; EPA: eicosapentaenoic acid; MSCs: mesenchymal stem cells; 9M5: furan fatty acid 9-(3-methyl-5-pentylfuran-2-yl)-nonanoic acid.

**Table 4:** Effect of Conjugated linoleic acid on PPARγ and PLIN1 modulation.

	Intervention	Duration	Study Design	Outcome	References
CLA	7.5 g/d of CLA or 8.78 g/d of palm oil as a control	3 weeks	In vivo Holstein cows *n* = 16	CLA decreases the mRNA abundance of genes related to fatty acid oxidation and lipolysis and increases the mRNA abundance of genes related to lipogenesis, such as PPARγ, in the adipose tissue of dairy cows	[64]
CLA	Supplementation 5 days/week with 600, 2000 or 3000 mg/kg of CLA or fish oil doses of 600 or 3000 mg/kg	4 weeks	In vivo BALB/c mice *n* = 30	Fish oil increases mitochondrial respiration in the liver. High doses of CLA produce steatogenic effects and promote lipid accumulation by downregulating PCG-1α	[65]
CLA	A basal diet with 1% of oleic acid, a diet supplemented with 0.5% of CLA and 1% of oleic acid or a diet supplemented with a 1% isomer-mix of CLA	8 weeks	In vivo V-line rabbits *n* = 75	Dietary supplementation of CLA produces a lower fat percentage via PPARγ regulation	[66]
CLA	Placebo sedentary/trained or CLA sedentary/trained (84% CLA, 12% oleic acid, 3% stearic acid, 0.5% palmitic acid, 0.5% linoleic acid)	6 weeks	In vivo BALB/c mice *n* = 32	CLA does not stimulate mitochondrial biogenesis or PCG-1α expression	[67]
CLA + α-lipoic acid	CLA at 0, 25 or 50 μM, α-lipoic acid at 0, 25 or 50 μM or a combination of both.	24 h	In vitro Murine macrophage RAW264.7 cells	CLA α-lipoic acid increases the expression of PPARγ and shows anti-inflammatory activity through ERK1	[68]

CLA: conjugated linoleic acid. Other fatty acids studied individually are listed in Table 5. Among them, sterol ester of α-linolenic acid stimulated mitochondrial biogenesis by PCG-1α regulation and reduced oxidative stress in a murine model with NAFLD [69]. However, in vitro studies showed the different effects of unsaturated fatty acids such as stearidonic and palmitoleic acids. While stearidonic acid inhibits adipocyte regulation by downregulating PPARγ protein, palmitoleic acid not only increased the transdifferentiation of bovine satellite cells into adipocytes by PPARγ but also upregulated PLIN1 proteins [70,71]. This is the only study to analyze the effect on PLIN1 protein expression. Regarding saturated fatty acids (SFA), the two studies analyzing the effect of palmitic acid demonstrated induced lipid accumulation and suppressed lipolysis by stimulating PPARγ protein in hepatic cells, promoting hepatotoxicity, and favored M1 macrophage polarization, enhancing TNF-α and IL-6 secretion and stimulating TLR4/ NF-κB signaling [72,73].

**Table 5:** Effect of different fatty acids on PPARγ and PLIN1 modulation.

	Intervention	Duration	Study Design	Outcome	References
Sterol ester of α-linolenic acid	Control diet, a high-fat diet rich in cholesterol or one based on plant sterol or ALA acid or sterol ester of ALA	16 weeks	In vivo C57BL/6J mice *n* = 50	Sterol ester of α-linolenic acid stimulates mitochondrial biogenesis by PCG-1α regulation and reduces oxidative stress in the NAFLD.	[69]
	0.9 mM of oleic acid, 0.1 mM of plant sterol, 0.1 mM of α-linolenic acid or 0.1 mM sterol ester of ALA	24 h	In vitro HepG2 cells		
Stearidonic acid	50 or 200 μM of stearidonic acid, EPA, DHA or ALA.	24, 75, and 144 h	In vitro 3T3-L1 preadipocytes	Stearidonic acid can inhibit adipocyte differentiation and reduce lipid accumulation by downregulating PPARγ	[70]
Palmitoleic acid (POA)	50 μM, 100 μM and 200 μM of POA	96 h	In vitro Bovine satellite cells (BSC)	All treatments of POA increased the protein expression of PPARγ and induced transdifferentiation of BSC into adipocytes. After treatment, PLIN1 is up-regulated	[71]
Palmitic acid	Palmitate at 50, 100 or 200 mM	24 h	In vitro HepG2 cells	Palmitate-induced lipid accumulation and suppressed lipolysis in HepG2 cells via PPARγ stimulation	[72]
Palmitic acid/ DHA	Normocaloric diet or high-fat diet with rosiglitazone	12 weeks	In vivo C57BL/6 mice	Palmitic acid induces the M1 polarized macrophage, which promotes lipid accumulation in hepatocytes via PPARγ and enhances the expression of IL-6 and TNF-α; DHA promotes M2 phenotype	[73]

ALA: α-linolenic acid; BSC: bovine satellite cells; HEPG2: human hepatocellular carcinoma cell line.

## Supplementary Materials

The following supporting information can be downloaded at: https://www.mdpi.com/article/10.3390/nu14224742/s1, Figure S1: Flowchart of studies through systematic review process.

## Abbreviations

9M5: furan fatty acid 9-(3-methyl-5-pentylfuran-2-yl)-nonanoic acid; ADRA2A: adrenoceptor alpha 2A; AGPAT2: 1-Acylglycerol-3-Phosphate O-Acyltransferase 2; AKT2: AKT serine/threonine kinase 2; ALA: α- linolenic acid; ARA: arachidonic acid; ART: antiretroviral therapy; AT: adipose tissue; BSCL2: lipid droplet biogenesis associated; CAV1: caveolin 1; CGL: congenital generalized lipodystrophy; CIDEC: cell death-inducing DFFA-like effector C; CLA: conjugated linoleic acid; DMT2: diabetes mellitus type 2; DHA: docosahexaenoic acid; EPA: eicosapentaenoic acid; FPLD: familial partial lipodystrophy; GLUT4: glucose transporter type 4; HIV: human immunodeficiency virus; IL-6: interleukin-6; KO: krill oil; LDL: low-density lipoprotein cholesterol; LIPE: lipase E; LMNA: lamin A/C; LO: linseed oil; MSCs: mesenchymal stem cells; NAFLD: non-alcoholic fatty liver disease; NFκB: nuclear factor kappa-light-chain-enhancer of activated B cells; NGR4: neuregulin 4; NLRP3: NLR family pyrin domain containing 3; NOS3: nitric oxide synthase 3; PBMCs: peripheral blood mononuclear cells; PD-1: programmed cell death protein 1; PGC-1α: peroxisome proliferator-activated receptor-γ coactivator 1α; PLIN 1: perilipin 1; PPARγ: peroxisome proliferator-activated receptor gamma; PUFA: n-3 polyunsaturated acid; SFA: saturated fatty acids; STRING 11: Search Tool for the Retrieval of Interacting Proteins 11; TLR: toll-like receptor; TNF-α: tumor necrosis factor alpha; UCP2: uncoupling protein 2; VLDLR: very-low-density lipoprotein receptor.
