# Supplementary figures and images for: PPARγ Gene as a Possible Link between Acquired and Congenital Lipodystrophy and its Modulation by Dietary Fatty Acids
Source: Nutrients. 2022 Nov 10;14(22):4742. doi: 10.3390/nu14224742 (PMC9693235; doi:10.3390/nu14224742)

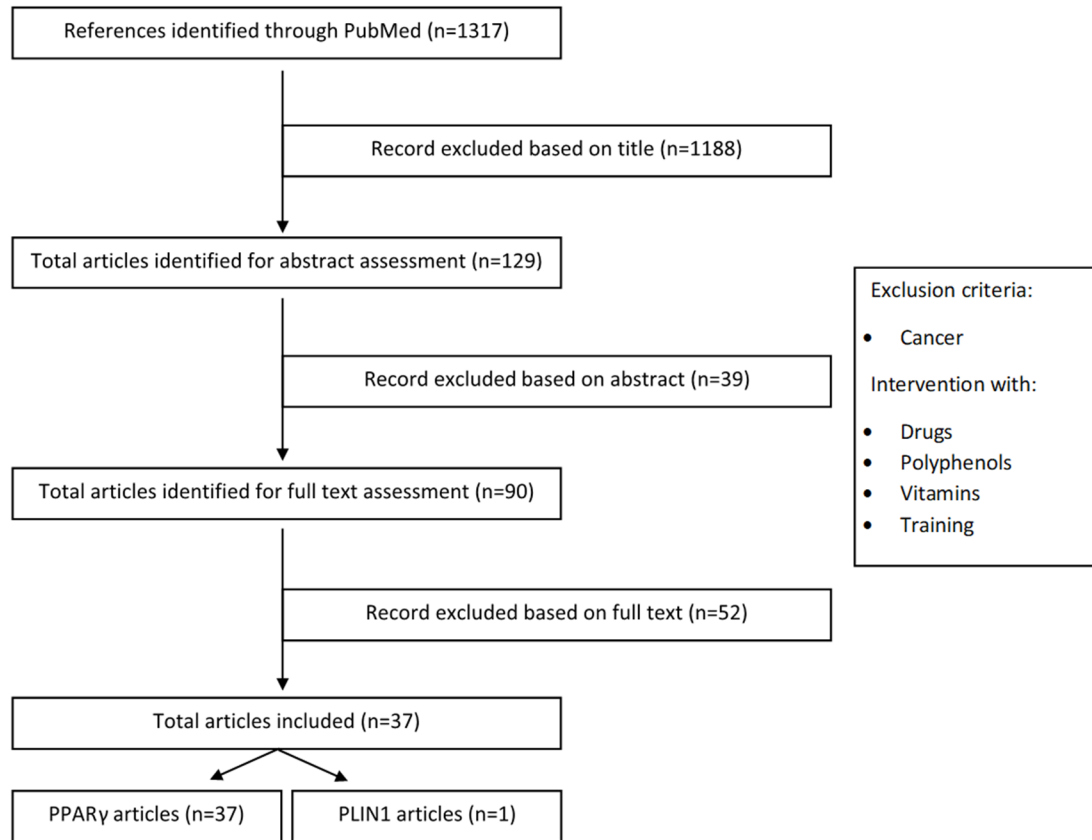

**Figure S1.** Flowchart of studies through systematic review process.

Supplement: Supplementary file 1 [file nutrients-14-04742-s001.zip › nutrients-1989702-supplementary.pdf]
